# A comparison of morning priming exercise using traditional-set and cluster-set configurations on afternoon explosive performance

**DOI:** 10.5114/biolsport.2024.133003

**Published:** 2024-01-02

**Authors:** Zhe Wang, Bing Yan, Yang Wang, Conghui Zhang, Feng Chen, Olivier Girard

**Affiliations:** 1 China Institute of Sport and Health Science, Beijing Sport University, Beijing, China; 2 School of Human Sciences (Exercise and Sports Science), The University of Western Australia, Perth, Western Australia, Australia

**Keywords:** Cluster sets, Priming resistance, Neuromuscular capabilities, Explosive strength, Jumping, Sprinting, Agility

## Abstract

The impact of two priming exercise protocols using traditional (TS) or cluster-set (CS) arrangements on explosive performance 6 hours later were examined. Sixteen male collegiate athletes performed three testing sessions (one baseline without any prior exercise in the morning and two experimental sessions) separated by 72 hours. Participants completed two morning (9–11 am) priming protocols in a randomized order, either using a TS (no rest between repetitions) or CS (30 seconds of rest between repetitions) configuration. The protocols consisted of 3 sets × 3 repetitions of barbell back squat at 85% of 1 repetition maximum, with 4 minutes of rest between sets. In the afternoon (3–5 pm) of each trial, after a 6-hour rest period, a physical test battery was conducted that replicated baseline testing, including countermovement jump, 20-meter straight-line sprint, and T-test abilities. Across both conditions, participants exhibited increased countermovement jump height, 20-meter sprint time and T-test time compared to baseline (P < 0.05). Improvements in countermovement jump height (+4.4 ± 5.4%; P = 0.008) and 20-meter sprint time (+1.3 ± 1.7%; P = 0.022), but not T-test time (+1.1 ± 3.3%; P = 0.585), were significantly greater for CS than TS. In conclusion, compared to a traditional set arrangement, a morning-based priming protocol using a cluster-set configuration led to superior explosive performance benefits in the afternoon.

## INTRODUCTION

Performing a low-volume resistance exercise, typically executed at maximal or near-maximal velocity, in the hours leading up to competition is a common priming exercise protocol [1]. According to a recent survey, external resistance workouts (e.g., jumping, squatting, pressing, and pulling derivatives) are often used in high performance sport to enhance afternoon explosive neuromuscular performance after completing the morning priming exercise [2]. Specifically, these delayed performance improvements have included enhanced force and power production during competitive actions, such as jumping [3, 4], sprinting [5, 6], or agility [5], after various priming exercises performed up to 48 h beforehand. While unloaded exercises and exercises utilizing elastic bands for external resistance are common loading strategies [1, 7], current evidence supports the use of resistance exercises, such as the back squat and bench press, performed in 2–4 sets of 3–5 repetitions at high resistance load (≥ 80% of 1 repetition maximum [1RM]) for lower body exercises [3, 5].

Literature indicates that morning priming exercise can improve afternoon explosive performance in swimmers [8], throwers [9], and rugby union players [6], while other studies have reported limited changes to countermovement (CMJ) performance following this practice [6, 10]. In the majority of available studies, the exercise set structure consisted of executing each repetition in sequence without rest between repetitions, known as a ‘traditional set’ structure [11]. One potential challenge with the traditional set (TS) structure is the ability to sustain high-velocity patterns throughout the session at least for moderate-to-high loads due to the accumulation of fatigue, which may be caused by decreased adenosine triphosphate and phosphocreatine availability, and increased lactate and/or ammonia accumulation [12]. This phenomenon is especially noticeable when lifting movements commonly used as priming exercises involve a small number of repetitions at higher loads in multiple sets [13].

To overcome the fatigue that comes with lifting heavier loads during priming exercises, one strategy is to adjust the set configuration [1, 7]. Cluster set (CS) structure is a technique that entails breaking down a set’s repetitions into smaller clusters with rest periods of ~20 to 40 seconds between them [11, 14]. Compared to a TS structure, a CS configuration may lead to better maintenance of movement velocity and power across sets and an entire exercise session [11, 15, 16, 17]. To date, CS arrangements in morning priming exercise literature have received limited attention, which has left many questions regarding their application and eventual benefits on explosive measures later in the day, including less frequently studied aspects such as sprinting and agility [7].

This study aimed to examine the impact of two priming exercise protocols, each featuring a different set arrangement (TS and CS), on performance measures including jumping, sprinting, and agility. It was hypothesized that a morning squat-based priming exercise utilizing a CS configuration would lead to greater enhancements in neuromuscular performance six hours after the end of the session compared to a TS structure.

## MATERIALS AND METHODS

### Participants

A repeated measures analysis of variance power calculation (α = 0.05, 1-β = 0.8) was conducted with G*Power (Version 3.1.9.3) to determine sample size based on one of our primary variables: sprint performance. The effect size of a priming exercise protocol on acute changes in sprint time, based on expected performance improvement after six hours of recovery, is 0.41 [5]. To express our results with 95% confidence, a minimum sample size of 15 participants was obtained. Therefore, to account for potential drop-outs or injuries, sixteen healthy males (age: 22.0 ± 2.16 years; height: 1.78 ± 0.05 m; body mass: 76.2 ± 8.3 kg) were recruited. Participants were classified as ‘Highly trained/National level’ (Tier 3) using established criteria [18], and were active in different sports (i.e., basketball, volleyball, track and field). They reported a two-year history of resistance training, including at least two sessions per week, and demonstrated experience in the back squat, with a minimum 1RM of 1.5 times their body mass (back squat 1RM: 146.1 ± 22.1 kg). The study was approved by Beijing Sport University Review Board for Human Participants (no. 2018018 H), and complied with the principles of the Declaration of Helsinki, with written informed consent obtained from participants.

### Study design

**FIG. 1:**
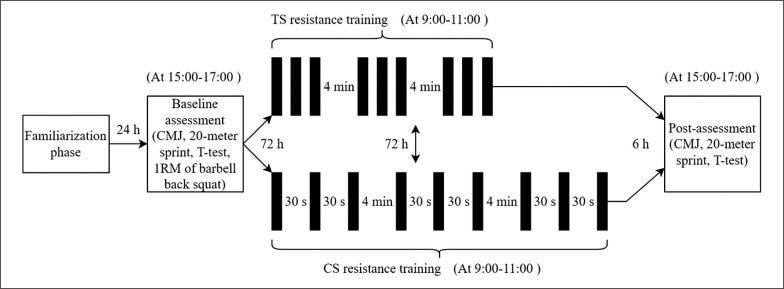
Study design. CMJ, countermovement jump. TS, traditional sets. CS, cluster sets.

The participants underwent five separate laboratory sessions, which included two familiarization sessions (visits 1 and 2), one baseline testing session (visit 3), and two main experimental sessions that were conducted in a randomized order (visits 4 and 5) over a period of three weeks. The visits were spaced 3–7 days apart. All participants were familiar with the testing procedures through their regular physical performance assessments in their clubs. Participants were instructed to avoid alcohol and caffeine consumption and refrain from strenuous training for 24 hours before each session. They were also asked to maintain a 24-hour food diary before the baseline testing session and to replicate their diet before the other two remaining trials. The participants were instructed to maintain their normal diet, avoiding nutritional supplements, during the testing protocol. Air temperature was maintained constant at ~22°C. An overview of the study design is depicted in Figure 1.

### Experimental visits

#### Familiarization sessions

During both sessions, participants visited the laboratory in the afternoon (3–5 pm), which coincided with the time of performance assessment during the two main experimental visits. The first step was to explain and demonstrate the detailed procedures and correct techniques for all the tests. Afterwards, participants practiced three sets of five repetitions of the barbell back squat with a light load (≤ 50% 1RM) on a Smith machine. To familiarize themselves with the test protocols and procedures, they performed a series of CMJ, 20-meter sprint, and T-test [3, 5, 19]. This included three repetitions of technique practices and three repetitions of maximal efforts, with ~3 minutes of recovery between each repetition.

### Baseline testing session

The baseline testing session was conducted in the afternoon (3–5 pm). Participants were first instructed to complete an 8-minute standardized warm-up routine, which consisted of five minutes of running and three minutes of dynamic stretching of hip and ankle mobility. Baseline values were then obtained for CMJ height, peak power, take-off velocity and push-off impulse, as well as 20-meter sprint performance and T-test performance. Each test was repeated three times, and the best trial was recorded for statistical analysis. Lastly, the 1 RM of the barbell back squat was determined.

### Main experimental sessions

Participants completed the standardized warm-up from the baseline testing session upon arriving at the laboratory. After a 5-minute rest in a passive seating position, they began a protocol consisting of three sets of three repetitions of barbell back squat at 85% of their 1RM on a Smith machine (Technogym Equipment, Italy). This was done in one of two experimental conditions: no rest between repetitions (TS) or a 30-second interval between repetitions (CS), with a 4-minute interval between sets in both cases [20]. After a 6-hour rest period, the testing routine was performed in a manner similar to the baseline testing session.

### Procedures

#### One-repetition maximum testing

Before the test performed on a Smith machine, participants completed a brief warm-up protocol consisting of submaximal squats at 50, 70, 80, and 90% of their self-estimated 1RM. This entailed 10, 6, 3 and 1 repetition(s) for each percentage, respectively, with a 2-minute rest between sets. Then, the load was gradually increased in 4–5 trials with at least 3 minutes of rest between each trial until the participant reached their 1RM [21]. The squat depth was individually standardized to parallel, meaning that they descended to reach a 90° knee angle. All participants reached their 1RM within a maximum of five trials.

### Countermovement jump test

To perform the CMJ test, participants were instructed to jump as high as possible. They started from a standing position and performed a downward countermovement to a self-selected depth, followed immediately by a rapid jump in one continuous movement. Participants were instructed to keep their hands on their hips to eliminate any influence of arm swing. The jumping was performed while participants stood on a force platform, which allowed for direct measurement of the vertical ground reaction forces. Data were sampled at a frequency of 1,000 Hz (model 9286BA, Kistler Corporation, Switzerland). Vertical jump height, peak power output, take-off velocity, and push-off impulse were all calculated using the MARS software (version 4.0; Kistler Corporation, Switzerland). Participants completed three trials of the CMJ, with a 1-minute rest between each trial. The mean of three trials was used for final analysis.

### 20-meter sprint test

To prepare for the 20-meter straight-line sprint test, participants underwent a 5-minute warm-up consisting of light drills, followed by 50% and 80% intensity efforts over the 20-meter distance. Participants started the test in a 2-point stance and then completed three maximal 20-meter sprints with a 2-minute walk-back recovery between each sprint. All tests were performed in a competitive manner, and the mean of three trials was used for final analysis. An automated light gate system (SmartSpeed™, Fusion Sport, Australia) with 0.01 s accuracy was used to record all times.

### T-test

The T-test involved arranging four cones in a T-shape, with one cone positioned 9.14 meter from the starting point and the other two cones placed 4.57 meter from either side of the second cone [22]. Participants were instructed to sprint 9.14 meter forward from the starting line to the first cone and touched it with their right hand. Then, they were required to move 4.57 meter to the left to reach the second cone and touch it with their left hand, followed by advancing 9.14 meter to the right to reach the third cone and touch it with their right hand. They then moved 4.57 meter to the left to the middle cone and touched it with their left hand before running back-ward to the starting line. The timing began with a sound signal and ended when the participant passed the timing gate when returning. If a participant failed to touch the designated cone, or crossed the legs while moving, or did not face forward throughout the testing period, the trial was considered unsuccessful. The timing was assessed using the same automated light gate system as for the 20-meter sprint.

### Statistical Analysis

Values are expressed as mean ± SD and 95% confidence interval (CI). For all dependent variables, the effect of condition was determined by a single factor analysis of variance for repeated measures (Baseline, TS, and CS). Data variance was first assessed using Mauchly test of sphericity, and a Greenhouse-Geisser correction was applied when required. *Post hoc* pairwise comparisons were performed using Bonferroni-adjusted P values. For each analysis of variance, partial eta-squared ($\eta_{\text{p}}^{2}$, with $\eta_{\text{p}}^{2}$ ≥ 0.06 representing a *moderate* effect and $\eta_{\text{p}}^{2}$ ≥ 0.14 a *large* effect) were calculated as measures of effect size. Cohen’s *d* effect sizes were calculated to determine meaningful differences, with *d* < 0.2, *d* = 0.2- < 0.5, *d* = 0.5–0.8 and *d* > 0.8 representing *trivial, small, moderate* and *large* effect sizes, respectively. To aid interpretation, the dependent variables were expressed as the percent change (%) from the Base-line measurements. Statistical significance was set at P ≤ 0.05.

## RESULTS

**FIG. 2:**
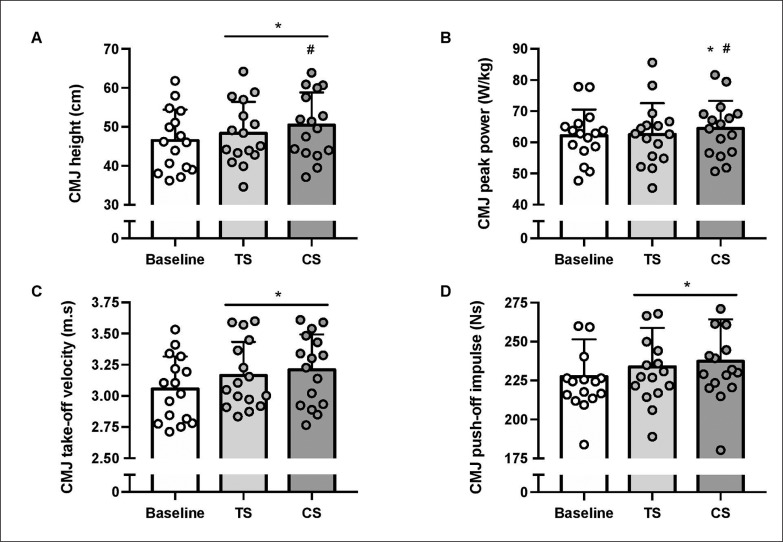
Countermovement (CMJ) height (A), peak power (B), take-off velocity (C), and push-off impulse (D) at baseline and six hours after a morning priming exercise protocol using traditional (TS) or cluster sets (CS). Values are Mean ± SD (n = 16). * and ^#^ significantly different from Baseline and TS, respectively.

Participants exhibited increased CMJ height in both TS (+4.1 ± 5.0%; P = 0.007) and CS (+8.7 ± 7.0%; P < 0.001) compared to Baseline, with CMJ height being higher in CS than TS (+4.4 ± 5.4%; P = 0.008) (Figure 2A; Table 1). Compared to Baseline, CMJ power output increased after CS (+3.6 ± 3.1%; P < 0.001), but not after TS (+0.4 ± 4.1%; P = 0.100). Additionally, CMJ power output was greater in CS than TS (+3.4 ± 4.4%; P = 0.040) (Figure 2B). Participants exhibited increased CMJ take-off velocity in both TS (+3.6 ± 3.7%; P = 0.004) and CS (+5.1 ± 3.8%; P < 0.001) compared to Baseline, with no significant differences between TS and CS (+1.5 ± 3.9%; P = 0.425) (Figure 2C). Regarding CMJ push-off impulse, both TS (+2.8 ± 3.1%; P = 0.006) and CS (+4.2 ± 2.7%; P < 0.001) resulted in improvements compared to Baseline, with no significant difference between TS and CS (+1.4 ± 2.7%; P = 0.128) (Figure 2D).

**TABLE 1:** Variables at baseline, traditional set (TS) and cluster sets (CS).

Variables	Conditions			ANOVA	Baseline – TS	Baseline – CS	TS – CS

	Baseline	TS	CS	*P value (* $\eta_{\text{p}}^{2}$ *)*	ES; MD*(95% CI)*	ES; MD*(95% CI)*	ES; MD*(95% CI)*
CMJ height (cm)	46.5 ± 7.9	48.3 ± 8.0*	50.4 ± 8.3*#	**P < 0.001*(0.58)***	**0.23**; -1.8*(-3.2;-0.5)*	**0.48**; -3.9*(-5.9;-2.0)*	**0.25**; -2.1*(-3.7;-0.5)*

CMJ peak power (W/kg)	62.18 ± 8.34	62.55 ± 10.01	64.42 ± 8.92*#	**P = 0.004*(0.34)***	0.04; -0.04(-3.54;-0.95)	**0.26**; -1.88*(-3.68;-0.07)*	**0.20**; 0.006*(-0.007;0.019)*

CMJ take-off velocity (m/s)	3.05 ± 0.26	3.16 ± 0.27*	3.21 ± 0.28*	**P < 0.001*(0.51)***	**0.41**; -0.11*(-0.18;-0.03)*	**0.57**; -0.05*(-0.13;0.03)*	0.17; 0.11*(-0.01;0.22)*

CMJ push-off impulse (Ns)	227.5 ± 23.8	233.8 ± 25.0*	237.3 ± 27.0*	**P < 0.001*(0.50)***	**0.25**; -6.3*(-10.9;-1.7)*	**0.37**; -9.7*(-15.4;-4.0)*	0.13; -3.4*(-7.5;0.7)*

20-m sprint time (s)	3.08 ± 0.12	3.01 ± 0.12*	2.97 ± 0.14*#	**P < 0.001*(0.61)***	**0.58**; 0.07*(0.02;0.12)*	**0.85**; 0.11*(0.07;0.15)*	**0.31**; 0.04*(0.01;0.07)*

T-test time (s)	10.39 ± 0.65	9.92 ± 0.64*	9.80 ± 0.53*	**P < 0.001*(0.54)***	**0.74**; 0.12*(0.16;0.79)*	**0.99**; 0.11*(0.29;0.88)*	0.20; 0.09*(-0.11;0.35)*

Values are Mean ± SD (n = 16). ES, effect size; MD, mean difference; 95% CI, 95% confidence interval.

* and # significantly different from Baseline and TS, respectively. Statistically significant ANOVA P values (partial eta-squared or $\eta_{\text{p}}^{2}$) and large effect sizes are listed in bold.

**FIG. 3:**
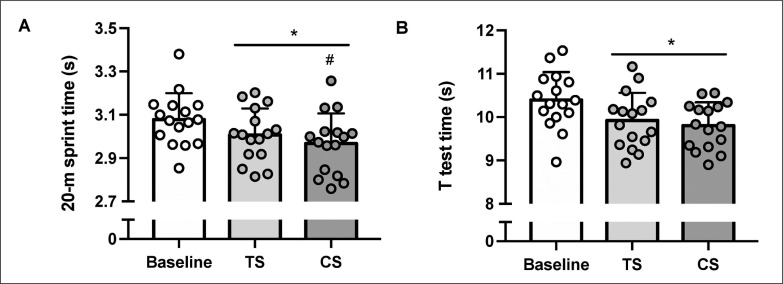
Performance time for 20-meter sprint (A) and T-test (B) at baseline and six hours after a morning priming exercise protocol using traditional (TS) or cluster sets (CS). Values are Mean ± SD (n = 16). * and ^#^ significantly different from Baseline and TS, respectively.

Both TS (+2.3 ± 2.5%; P = 0.007) and CS (+3.6 ± 2.2%; P < 0.001) resulted in faster sprint times compared to Baseline, with also faster sprint times in CS than TS (+1.3 ± 1.7%; P = 0.022) (Figure 3A; Table 1). Faster T-test times were observed after both TS (+4.5 ± 4.4%; P = 0.003) and CS (+5.5 ± 4.0%; P < 0.001) compared to Baseline, with no significant difference between TS and CS (+1.1 ± 3.3%; P = 0.585) (Figure 3B; Table 1).

## DISCUSSION

Our intention was to compare the effects of two priming exercise protocols using low-volume back squats, with either a TS or CS configuration, on explosive performance after six hours of rest. Taken together, and in agreement with our hypothesis, a morning priming exercise improved jumping, sprinting, and agility performance in the afternoon. However, the CS configuration, with 30 seconds of rest between repetitions, was a better strategy to maximize neuromuscular performance compared to the TS arrangement, which had no rest within the sets.

A major finding of our study was the superior jumping and sprinting performance observed with the CS protocol. Arguably, the rest intervals incorporated in the CS configuration may have contributed to a better potentiation/fatigue balance [15], thus enabling greater performance benefits later in the day. This suggestion is consistent with previous research on post-activation performance enhancement, which has shown that CS with heavy loads (≥ 85% 1RM) results in better performance compared to TS arrangements [20, 23]. It is noteworthy that in these studies, performance evaluation was limited to only a few minutes after the intervention. For instance, Nickerson et al. [20] showed a significantly greater post-activation performance enhancement effect in the 20-meter sprint induced by CS compared to TS. From a neuromuscular perspective, high-frequency motor neuron activation, mechanical stiffness, and contractile properties are potential mechanisms for performance facilitation in the minutes after the intervention [24]. However, there is still uncertainty regarding the role of the neuromuscular system in priming that occurs six hours before performance assessment [7].

Another key finding was that CMJ peak power improved only in CS. Both TS and CS protocols also resulted in enhancements in CMJ height, take-off velocity, and push-off impulse six hours after the priming exercise. Additionally, participants sprinted faster following priming exercise protocols. These observations are consistent with several studies showing that high-intensity, low-volume priming exercise can induce positive effects for CMJ and/or 40-meter sprint performance due to enhanced neuromuscular efficacy [5, 6, 25, 26]. For example, semi-professional rugby players who engaged in priming exercise with an intensity ranging from 80–100% 1RM showed improvements in 3RM bench press, 3RM back squat, and CMJ peak power, as well as a decrease in 40-meter sprint time [5]. In our study, both TS and CS protocols led to comparable benefits (i.e., 4.5% *vs*. 5.5%, respectively) for agility performance relative to baseline. However, no previous studies were found that compared the afternoon performance changes induced by morning CS and TS priming exercises, limiting potential comparisons with the literature. Agility is largely determined by strength and power production capabilities [27], which probably explains why the improved T-test times were consistent with beneficial changes in CMJ and 20-meter sprint performance.

In this study, most athletes showed a positive response to moderate exercise volumes, while others did not. This raises the question of what the minimum effective dose should be for each athlete to achieve significant performance gains. It is argued that when heavy loads (≥ 80% 1RM) are used, an appropriate exercise volume (reps × sets × % 1RM) is necessary to induce beneficial effects [7]. In our study, both conditions had a total exercise volume of 765 a.u, with the same number of repetitions performed. This falls within the recommended dose range of 760–1190 a.u [7]. However, visual examination of Figures 2 and 3 indicates a wide range of inter-individual responses, which may be partly attributed to methodological factors. Firstly, the chronotype of participating athletes was not assessed, nor were their workout preferences in terms of their habitual precompetition strategies (i.e., exercise selection and resistance loading methods) monitored. Secondly, the athletes tested may have been at different stages of their training cycles, ranging from periods of detraining and rest to their peak competitive state. This variation in training could have influenced their physical and mental approach to the testing process.

There are several methodological and logistical aspects that need to be emphasized. While the two different priming sessions were randomized, a primary limitation of this study is the lack of randomization regarding the order in which participants completed experimental protocols and baseline testing. Additionally, neurophysiological variables such as surface EMG indicators, as well as hormonal variables such as testosterone concentration, were not measured. Previously, both serum testosterone and cortisol levels were elevated six hours after a morning-based strength exercise session [5]. However, it remains to be investigated whether the morning CS workout condition resulted in higher blood testosterone levels later in the day compared to using TS arrangements.

An additional consideration is the assessment of athletes in the afternoon at a single time point. However, delayed performance benefits have been observed after various priming exercises performed up to 48 hours beforehand [1, 4, 7, 26]. Therefore, future studies should investigate the effects of our priming exercise protocols at different time points to determine the optimal recovery time for prescribing priming exercise. While there is growing interest in CS paradigms, it is still uncertain which method of CS application is more effective in enhancing neuromuscular performance. This includes the manipulation of rest periods between clusters of repetitions, as done in our study, or after each individual repetition within a set [15]. Investigation into different CS configurations could add further value for researchers and practitioners alike.

### Practical applications

The majority of the sixty-nine practitioners (~84%) surveyed by Harrison et al. [2] believed that priming exercise in high performance sport is beneficial for enhancing performance. Our study results support this idea and add that including a barbell back squats session utilizing a CS configuration (30-second interval between repetitions, 4-minute interval between sets) six hours prior to testing can be an effective ergogenic strategy for improving explosive performance compared to a no morning exercise condition. The cost of using a CS configuration is negligible, especially considering performance improvements observed herein, and the fact that it only adds a few extra minutes to the completion time. When competitions are scheduled in the afternoon, strength and conditioning coaches could therefore prescribe a high-load, low-volume squat-based priming exercise (85% 1RM, three sets of three repetitions) in the morning to enhance jumping, sprinting, and agility in the afternoon.

## CONCLUSIONS

The effects of two morning priming exercise protocols, both involving low-volume back squats, on explosive performance after six hours of rest were compared: one with a TS (nor rest between repetitions) configuration and the other with a CS (30 seconds of rest between repetitions) configuration. Compared to TS, employing a CS configuration further enhances performance benefits later in the day, resulting in improvements of 1–4.5%.

## References

[1] Mason B, McKune A, Pumpa K, Ball N. The use of acute exercise interventions as game day priming strategies to improve physical performance and athlete readiness in team-sport athletes: a systematic review. Sports Med. 2020; 50(11):1943–1962. 32779102 10.1007/s40279-020-01329-1

[2] Harrison PW, James LP, McGuigan MR, Jenkins DG, Kelly VG. Prevalence and application of priming exercise in high performance sport. J Sci Med Sport. 2020; 23(3):297–303. 31594712 10.1016/j.jsams.2019.09.010

[3] Saez Saez de Villarreal E, González-Badillo JJ, Izquierdo M. Optimal warm-up stimuli of muscle activation to enhance short and long-term acute jumping performance. Eur J Appl Physiol. 2007; 100(4):393–401. 17394010 10.1007/s00421-007-0440-9

[4] Tsoukos A, Veligekas P, Brown LE, Terzis G, Bogdanis GC. Delayed effects of a low-volume, power-type resistance exercise session on explosive performance. J Strength Cond Res. 2018; 32(3):643–650. 28291764 10.1519/JSC.0000000000001812

[5] Cook CJ, Kilduff LP, Crewther BT, Beaven M, West DJ. Morning-based strength training improves afternoon physical performance in rugby union players. J Sci Med Sport. 2014; 17(3):317–321. 23707139 10.1016/j.jsams.2013.04.016

[6] Russel M, King A, Bracken RM, Cook CJ, Giroud T, Kilduff LP. A comparison of different modes of morning priming exercise on afternoon performance. Int J Sports Physiol Perform. 2016; 11(6):763–767. 26658460 10.1123/ijspp.2015-0508

[7] Harrison PW, James LP, McGuigan MR, Jenkins DG, Kelly VG. Resistance priming to enhance neuromuscular performance in sport: evidence, potential mechanisms and directions for future research. Sports Med. 2019; 49(10):1499–1514. 31203499 10.1007/s40279-019-01136-3

[8] McGowan CJ, Pyne DB, Thompson KG, Raglin JS, Rattray B. Morning exercise: enhancement of afternoon sprint-swimming performance. Int J Sports Physiol Perform. 2017; 12(5):605–611. 27617694 10.1123/ijspp.2016-0276

[9] Ekstrand LG, Battaglini CL, McMurray RG, Shields EW. Assessing explosive power production using the backward overhead shot throw and the effects of morning resistance exercise on afternoon performance. J Strength Cond Res. 2013; 27(1):101–106. 22395279 10.1519/JSC.0b013e3182510886

[10] Woolstenhulme MT, Bailey BK, Allsen PE. Vertical jump, anaerobic power, and shooting accuracy are not altered 6 hours after strength training in collegiate women basketball players. J Strength Cond Res. 2004; 18(3):422–425. 15320641 10.1519/13463.1

[11] Haff GG, Hobbs RT, Haff EE, Sands WA, Pierce KC, Stone MH. Cluster Training: A novel method for introducing training program variation. Strength Cond J. 2008; 17(1):67–76.

[12] Gorostiaga EM, Navarro-Amezqueta I, Calbet JA, et al. Blood ammonia and lactate as markers of muscle metabolites during leg press exercise. J Strength Cond Res. 2014; 28:2775–2785. 24736776 10.1519/JSC.0000000000000496

[13] Benjanuvatra N, Bardbury D, Landers G, Goods P, Girard O. How does multi-set high-load resistance exercise impact neuromuscular function in normoxia and hypoxia? Eur J Sport Sci. 2022; 17(5):658–665. 10.1080/17461391.2022.2095929 35770524

[14] Tufano JJ, Brown LE, Haff GG. (2017). Theoretical and practical aspects of different cluster set structures: a systematic review. J Strength Cond Res. 2017; 31(3):848–867. 27465625 10.1519/JSC.0000000000001581

[15] Latella C, Teo WP, Drinkwater EJ, Kendall K, Haff GG. The acute neuromuscular responses to cluster set resistance training: a systematic review and meta-analysis. Sports Med. 2019; 49(12):1861–1877. 31506904 10.1007/s40279-019-01172-z PMC6851217

[16] Tufano JJ, Conlon JA, Nimphius S, Brown LE, Seitz LB, Williamson BD, Haff GG. Maintenance of velocity and power with cluster sets during high-volume back squats. Int J Sports Physiol Perform. 2016; 11(7):885–892. 26791936 10.1123/ijspp.2015-0602

[17] Dello Iacono A, Beato M, Halperin I. The effects of cluster-set and traditional-set postactivation potentiation protocols on vertical jump performance. Int J Sports Physiol Perform. 2019; 14(1):74–79. 10.1123/ijspp.2019-0186 31614331

[18] McKay AK, Stellingwerff T, Smith ES, Martin DT, Mujika I, Goosey-Tolfrey VL, Sheppard J, Burke L. Defining training and performance caliber: a participant classification framework. Int J Sports Physiol Perform. 2022; 17(3):317–331. 34965513 10.1123/ijspp.2021-0451

[19] Nimphius S, McGuigan MR, Newton RU. Relationship between strength, power, speed, and change of direction performance of female softball players. J Strength Cond Res. 2010; 24(4):885–895. 20300038 10.1519/JSC.0b013e3181d4d41d

[20] Nickerson BS, Mangine GT, Williams TD, Martinez IA. Effect of cluster set warm-up configurations on sprint performance in collegiate male soccer players. Appl Physiol Nutr Metab. 2018; 43(6):625–630. 29365286 10.1139/apnm-2017-0610

[21] Dæhlin TE, Haugen OC, Haugerud S, Hollan I, Raastad T, Rønnestad BR. Improvement of ice hockey players’ on-ice sprint with combined plyometric and strength training. Int J Sports Physiol Perform. 2017; 12(7):893–900. 27918670 10.1123/ijspp.2016-0262

[22] Pauole K, Madole K, Garhammer J, Lacourse M, Rozenek R. Reliability and validity of the T-Test as a measure of agility, leg power, and leg speed in college-aged men and women. J Strength Cond Res. 2000; 14(4):443–450.

[23] Boullosa DA, Abreu L, Beltrame LG, Behm DG. The acute effect of different half squat set configurations on jump potentiation. J Strength Cond Res. 2013; 27(8):2059–2066. 23207892 10.1519/JSC.0b013e31827ddf15

[24] Blazevich AJ, Babault N. Post-activation potentiation versus post-activation performance enhancement in humans: historical perspective, underlying mechanisms, and current issues. Front Physiol. 2019; 10:1359. 31736781 10.3389/fphys.2019.01359 PMC6838751

[25] Fry AC, Stone MH, Thrush JT, Frazee SJ. Precompetition training sessions enhance competitive performance of high anxiety junior weightlifters. J Strength Cond Res. 1995; 9(1):37–42.

[26] González-Badillo JJ, Rodríguez-Rosell D, Sánchez-Medina L, Ribas J, López-López C, Mora-Custodio R, Yañez-García JM, Pareja-Blanco F. Short-term recovery following resistance exercise leading or not to failure. Int J Sports Med. 2016; 37(4):295–304. 26667923 10.1055/s-0035-1564254

